# Effects of COVID-19 pandemic on low birth weight in a nationwide study in India

**DOI:** 10.1038/s43856-024-00545-4

**Published:** 2024-06-14

**Authors:** Santosh Kumar, Clare Hill, Timothy J. Halliday

**Affiliations:** 1 Keough School of Global Affairs, University of Notre Dame, Notre Dame, IN 46556 USA; 2 Department of Economics, University of Hawaii, Honolulu, HI 96822 USA

**Keywords:** Public health, Preterm birth

## Abstract

**Background:**

Among newborns, those born in India have the highest prevalence of low birth weight (LBW). The COVID-19 pandemic exacerbated the risk factors for LBW. This study examined whether birth outcomes deteriorated during the pandemic period compared to those during the pre-pandemic period.

**Methods:**

This cross-sectional study included nationally representative data on 198,203 infants. Multivariate ordinary least square and logistic regression models with district fixed effects were fitted to compare the birth outcomes in the pandemic period (April 2020-April 2021) and the pre-pandemic period (July 2014-December 2019). Regression models were adjusted for covariates—gender and birth order of the child, mother’s age and education, rural residence, religion, caste, and household wealth.

**Results:**

The pandemic cohort includes 11,851 infants (5.8%), while the pre-pandemic cohort includes 192,764 infants (94.2%). The LBW prevalence rate is 20% in the pandemic cohort and 17% in the pre-pandemic cohort. The covariate-adjusted model shows significant differences in birth weight (11 grams) and LBW (aOR: 1.08; 95% CI: 1.03-1.14) between the pandemic and pre-pandemic cohorts.

**Conclusions:**

Our findings show that babies born during the COVID-19 pandemic are more likely to be LBW. The subgroup analyses indicate significant differences by religion but not by maternal education, caste, and wealth group.

## Plain language summary

Globally, approximately 20 million babies are born with low birth weight (weighing less than 2,500 grams) every year. These babies are at increased risk of physical and cognitive deficits and behavioral abnormalities in their later lives. In this research, we investigate the impact of the COVID-19 pandemic on the prevalence of low birth weight in India. Our findings reveal that babies born between April 2020 and April 2021 faced a higher risk of being born as low birth weight compared to those born before April 2020. This underscores the necessity for targeted policies aimed at mitigating the risk of low birth weight during periods of high COVID-19 infection, especially for vulnerable populations in low and middle-income countries.

## Abstract (web summary)

Kumar et al. examine differences in birthweight and LBW between the pre COVID-19 (before Jan 2020) and COVID-19 (after March 2020 onwards). Infants born during the COVID-19 period had lower birthweight compared to infants born during pre-COVID-19 periods.

## Introduction

The COVID-19 pandemic has had adverse impacts on the delivery of essential maternal and neonatal health services globally. Lockdowns, travel restrictions, and fear of infection made it difficult for many women to access prenatal care, delivery services, and postpartum care. This led to an increase in home births and a reduction in facility-based births, which increased the risk of maternal and neonatal morbidity and mortality. This may have led to adverse impacts on birth weight, a major determinant of infant health and survival.

Globally, one in every four (approximately 23.4%) or 30 million babies are born as low birth weight (LBW—birth weight <2500 g) infants, and approximately 95% of these LBW births occur in low- and middle-income countries^1^. It is estimated that up to 27% of all births are born as LBW in South Asia, and approximately 50% of all LBW babies are born in South Asia^2^. In addition to increasing the risk of morbidity and mortality among infants, LBW also has long-term implications for human capital development in resource-constrained countries. LBW babies are more likely to experience developmental delays, cognitive impairments, and behavioral problems, which can impact their ability to learn and succeed in school^3^. LBW infants accumulate less human capital, which affects their employment opportunities, labor force participation, and wages later in life^4,5^.

The COVID-19 pandemic affected over 181 million individuals worldwide and killed 3.9 million people worldwide as of June 27, 2021^6^. The pandemic affected all parts and all ages of the Indian population, but the older population as well as populations with chronic medical conditions such as diabetes and hypertension were at greater risk of contracting COVID-19. As of January 2023, there were more than 44 million confirmed cases of COVID-19 and half a million COVID-19 deaths in India^7^. The Indian government announced a nationwide lockdown on 22nd March 2020 to address the unprecedented increase in the number of COVID-19 cases. Given the substantially greater incidence of LBW and the severe health impacts of the COVID-19 pandemic, a critical policy-relevant question is to examine the effects of the COVID-19 pandemic on birth outcomes in an under-resourced setting.

The empirical evidence on the adverse effects of COVID-19 lockdowns is limited and mixed. Maternal stress and reduced prenatal care during the pandemic could increase preterm birth and LBW rates^8^. In contrast, several studies either found no effects or the opposite association between the pandemic and LBW rates. In richer settings such as Ireland, Denmark, Austria, and Turkey, studies have shown substantial reductions in the rate of very low birth weight^9–12^. These studies showed an increase in mean birth weight, lower rates of low birth weight, and very low birth weight during the lockdown period compared to the pre-lockdown period.

On the other hand, a few studies either found no effects of lockdowns or found a decrease in birth weight. An Argentinian study comparing births in public versus private hospitals found no significant difference in birth weight between the pandemic and pre-pandemic cohorts^13^. The risks for both LBW and small for gestational age increased over the COVID-19 period in China and Korea^14,15^.

In this study, we assess the effect of the COVID-19 pandemic on the incidence of LBW in India. Although prior studies have examined the impacts of the pandemic on LBW in other countries, there is a dearth of similar studies conducted specifically in India. We examine differences in birthweight and LBW between the pre-pandemic (before Jan 2020) and pandemic years (after March 2020 onward). We use nationally representative household data to test the hypothesis that the mean birth weight decreased during the pandemic years; thus, neonates born during this period may have had a greater prevalence of LBW than those born during the pre-pandemic period. Additionally, we examine the associations between LBW incidence and COVID-19 incidence according to maternal education, household wealth, religion, and caste. We find that babies born between April 2020 and April 2021 are at greater risk of LBW after we adjusted for sociodemographic covariates and district fixed effects.

## Methods

### Data and variables

We use data from the fifth round of the Indian National Family Health Survey (NFHS-5). The NFHS data are publicly available and can be downloaded from the Demographic and Health Survey website https://dhsprogram.com). Access to the NFHS data is granted for research purposes after registering as an NFHS data user. We registered with the DHS website to gain access to the dataset. The reporting in this cross-sectional study followed the Strengthening the Reporting of Observational Studies in Epidemiology (STROBE) guidelines. We used publicly available, anonymous, secondary data; thus, ethical approval was not required for the current study. The ICF’s IRB reviewed our project information form and supporting materials submitted as part of the DHS/2019–2021 India National Family Health Survey and determined that the activities described are not human subjects research (NHSR) and thus waived the IRB for this study.

The NFHS-5 survey was conducted during 2019–2021. The NFHS data are representative at the district level and provide information on population, health, and nutritional outcomes for each district in India. The NFHS rolled out four survey modules: household, women, men, and biomarkers. The survey provides detailed data on the socioeconomic characteristics of the household, access and use of health services, maternal and child health, fertility history, nutrition, etc.

The NFHS-5 survey was conducted in two phases, phase one from 17th June 2019 to 30th January 2020 and phase two from 2nd January 2020 to 30th April 2021. The sample included 636,699 households, 724,115 women, and 101,839 men drawn from all districts in India. A two-stage sample selection design was adopted. In rural areas, villages/communities/clusters were selected with a probability proportional to their size. In the second stage, 22 households were selected randomly. Similarly, Census Enumeration Blocks (CEB) formed the primary sampling unit in urban areas, and 22 households were selected randomly from each CEB. The sample was drawn from 707 districts in 36 states and union territories (UTs).

The two phases of data collection in the pre-pandemic and pandemic periods are well suited for estimating the effects of the COVID-19 pandemic on birth outcomes. The first phase of data collection was conducted before the pandemic, while the second phase collected data during the pandemic period. The primary outcomes included in the analysis were birth weight (measured in grams) and a binary indicator of LBW. The NFHS survey recorded birth weight information either from the birth card or from the mother’s recall. Birth weight data were available for 90% of births (IIPS & ICF, 2021); these birthweight data were collected from the birth card for 60% of children, while the birthweight data for the remaining 40% were based on the mothers’ recall. Although recall bias may be an issue, the correlation between birth weight when recalled by the mother and when measured by a medical professional was 0.9 across 19 studies in a recent meta-analysis, indicating that the mother’s recall of birth weight is a good predictor of the actual birth weight^16^. However, recall bias does appear to be lower in developed countries.

The main exposure variable is whether births occurred in the first wave of the pandemic—children born between April 2020 and April 2021 were defined as the pandemic or COVID-19 cohort (*n* = 11,851). In contrast, children born before 2020 (July 2014–December 2019) were defined as the pre-pandemic or non-COVID-19 cohorts (*n* = 192,764). We excluded births in January and March 2020 to eliminate the partially exposed cohort. The pandemic cohort is coded as one, while the pre-pandemic group is coded as zero and serves as the reference group.

The pregnancy details were available for 232,920 children in the survey. Of these, 19,358 (8.3%) children were not weighed at birth, and 4296 (1.8%) children had no birth weight information. After excluding the outliers and restricting the sample to the plausible birth weight range of 500–5000 g, the BW sample included 208,950 children. We used listwise deletion to exclude observations with missing values for the control variables. Exclusion of these observations may lead to sample selection bias depending on whether missingness is random or non-random. If the data are missing completely at random, then the listwise deletion does not bias the estimated regression coefficients. In the case of non-random missing data, the regression coefficients are biased. In our sample, the majority of the missing data are on the dependent variable (birth weight), and since we are estimating logistic regression for LBW outcomes, listwise deletion still provides approximately unbiased estimates of the regression coefficients for the population for whom we have birth weight information. We prefer listwise deletion to handle the missing observations because it allows for the correct standard errors and reflects the actual amount of information used in the analysis. The final analytical sample after excluding observations with missing values includes 204,615 children.

### Statistical analysis

Categorical variables are reported as percentages and continuous variables are reported as the means. We calculated the means for the variables separately for the pandemic and pre-pandemic cohorts. A 2-sided significance level at the 5% level was used to test for differences between the pandemic and pre-pandemic cohorts. Multivariate linear models were estimated via ordinary least squares (OLS) for the birth weight outcome, and multivariate logistic models were estimated via Maximum Likelihood Estimation for the LBW outcome. When estimating logistic models, we calculated the odds of LBW for children who were born during the pandemic period compared with those who were born before the pandemic. We report β coefficients from the OLS models and odds ratios (ORs) from the logistic models.

To minimize biases from potential confounding variables, we adjusted for gender and birth order of the child, household caste, religion of the household (the Hindu religion is coded as one), rural residence, mother’s age and education, and a wealth index for the household. We cluster standard errors at the district level and include district fixed effects to adjust for district-specific time-invariant characteristics that may affect birth outcomes such as the district’s health infrastructure and level of development. All analyses were performed in Stata, version 17^17^. ORs are reported with 95% CIs.

### Reporting summary

Further information on research design is available in the Nature Portfolio Reporting Summary linked to this article.

## Results

### Summary statistics

Table 1 shows the socioeconomic characteristics of the pandemic and pre-pandemic cohorts. The analytical sample includes 204,615 children born between 2014 and 2021. Approximately 8% of the children were in the pandemic group, while the remaining 92% of the children were in the pre-pandemic group. Pre-pandemic children were born between July 2014 and December 2019, while pandemic cohorts were born between April 2020 and April 2021. There were statistically significant differences in BW and the prevalence of LBW between the pandemic and pre-pandemic groups. Pandemic-affected children had lower BW (45 g difference) and a greater probability of being born as LBW (20% vs. 17%, *p* < 0.001, Table 1).

**Table 1:** Summary statistics (*N*: 204,615)

	Pandemic cohort	Prepandemic cohort	Pandemic–prepandemic cohort	
	Mean/%	Mean	Difference	*P*-value
	(1)	(2)	(3)	(4)
Birth weight (g)	2763.32	2809.98	−46.65	5 × 10^−19^
LBW (<2.5 kg)	20%	17%	0.03	7 × 10^−10^
Female	49%	48%	0.007	0.084
Birth order	2.05	2.08	−0.04	0.028
Mother’s age	25.52	27.36	−1.83	0.00008
*Maternal education*				
Years of schooling	8.47	7.79	0.68	1 × 10^−44^
No education	18%	20%	−0.02	3 × 10^−8^
Primary completed	10.4%	13%	−0.02	6 × 10^−13^
Secondary completed	53%	53%	−0.001	0.686
Higher than secondary	18.7%	14%	0.04	3 × 10^−31^
SCST	43%	39%	0.03	3 × 10^−13^
Hindu religion	82%	74%	0.08	1 × 10^−94^
Rural	80%	79%	0.02	4 × 10^−6^
*Wealth quintiles*				
Poorest	27%	24%	0.02	2 × 10^−12^
Poorer	21%	23%	−0.02	8 × 10^−10^
Middle	18%	20%	−0.02	3 × 10^−19^
Richer	17%	18%	−0.006	0.011
Richest	17%	14%	0.03	1 × 10^−12^
Observations	11,851 (7.8%)	192,764 (92.2%)		

Notes: Pandemic cohorts are children who were born between April 2020 and April 2021. Pre-pandemic cohorts were born between July 2014 and December 2019. Means and proportions are reported for continuous and categorical variables, respectively. SCST are scheduled caste and scheduled tribes which are socially and economically disadvantaged communities. Maternal education is categorized as no education, primary education, secondary education, and higher than secondary education. Household wealth quintiles are categorized as poorest, poor, middle, richer, and richest groups.

There are statistically significant differences in household characteristics between the two groups. Specifically, mothers of the pandemic cohort are younger and have more years of schooling, and a greater proportion of pandemic-affected children come from rural areas, socially disadvantaged communities of scheduled caste and tribe, and Hindu households. The other major difference was that a greater proportion of the pandemic cohort was from the poorest (27% vs. 24%, *p* < 0.001, Table 1) and richest wealth groups (17% vs 14%, *p* < 0.001, Table 1).

### Association between pandemic exposure and birth outcomes

Table 2 shows the associations between birth outcomes—BW and the probability of LBW—and exposure to COVID-19 during the pandemic. In all estimations, we adjusted for the potential confounders mentioned above. In column 1, we estimate the model using OLS, while in column 2, we estimate the model using logistic regression (odds ratios are reported). Births during the pandemic years are associated with a lower BW of 11 g after we adjust for district fixed effects (col. 1). Gender, scheduled caste/scheduled tribe (SCST), and Hindu religion were negatively associated with BW, while birth order, maternal age, and rural residence were positively associated with BW. SC/STs are scheduled caste and scheduled tribes that are socially and economically disadvantaged communities.

**Table 2:** Association between COVID-19 period and birthweight, *n* = 204,615 (fixed-effects models)

	BW	LBW
	(β)	AOR (95% CI)
	(1)	(2)
Pandemic cohort	−11.0**	1.08
	(5.52)	(1.03–1.14)
Gender (female=0)	−62.62***	1.18
	(2.39)	(1.15–1.20)
Birth order	9.88***	0.97
	(1.31)	(0.95–0.98)
Scheduled caste/tribe	−8.95***	1.003
	(2.90)	(0.97–1.03)
Hindu religion	−33.99***	1.08
	(3.74)	(1.03–1.13)
Mother’s age	2.88***	0.99
	(0.31)	(0.98–0.99)
Rural residence	11.77***	0.94
	(3.64)	(0.91–0.98)
*Maternal education*		
No education	*Ref*.	*Ref*.
Primary	−0.22	1.02
	(4.59)	(0.97–1.06)
Secondary	31.57***	0.91
	(3.80)	(0.88-0.95)
Higher	87.22***	0.76
	(5.18)	(0.72–0.80)
*Wealth quintiles*		
Poorest	*Ref*.	*Ref*.
Poor	34.03***	0.89
	(3.73)	(0.86–0.92)
Middle	70.12***	0.77
	(4.21)	(0.74–0.81)
Rich	94.58***	0.72
	(4.76)	(0.69–0.76)
Richest	126.22	0.65
	(5.77)	(0.61–0.69)
District fixed effects	Yes	Yes

Notes: *CI* confidence intervals, AOR adjusted odds ratios, BW birthweight, LBW low birth weight, LBW (birthweight <2500 g). Robust standard errors are reported in parentheses in columns, while 95% CIs are reported in parentheses in column 2. Pandemic cohorts are children born between April 2020 and April 2021. Maternal education is categorized as no education, primary education, secondary education, and higher than secondary education. Household wealth quintiles are categorized as poorest, poor, middle, richer, and richest groups.

***, ** denotes statistical significance at 1% and 5% level of significance, respectively.

We find a significant wealth and education gradient with children born to educated mothers and in wealthier households having higher BW compared to the children born in the reference groups. We further tested the sensitivity of the results by estimating a random-effects model (Supplementary Table S1). The results from fixed-effects and random-effects models are similar, thus lending credence to the main findings that pandemic cohorts had lower birthweights than pre-pandemic cohorts.

The results from logistic regression models show that compared with prepandemic cohorts, pandemic cohorts have 1.08 times greater odds (95% CI, 1.03–1.14) of having LBW. Compared with those of the poorest households, the odds of LBW are lower for children born to highly educated mothers and in households in the top four wealth groups.

### Subgroup results by mother’s education, wealth group, caste, and religion

**Fig. 1:**
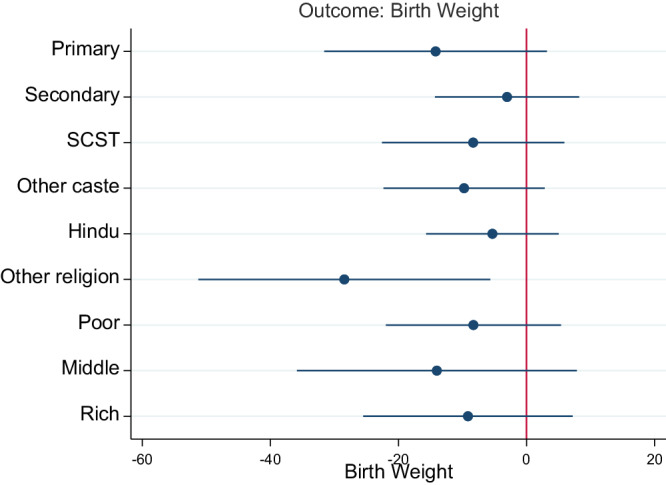
Stratified analyses by mother’s education, caste, religion, and household wealth (birth weight). Notes: Coefficients from linear regression are reported, the bar shows 95% CI. All models adjust for the confounders and district fixed effects reported in Table 2. BW is reported in grams. SCST are scheduled caste and scheduled tribes which are socially and economically disadvantaged communities.

**Fig. 2:**
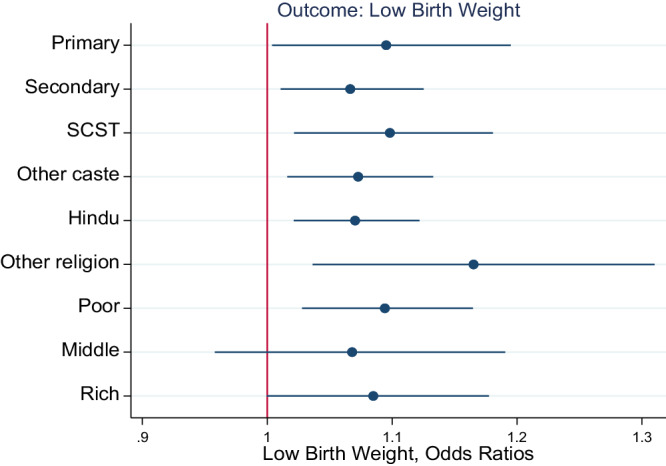
Stratified analyses by mother’s education, caste, religion, and household wealth (Low birth weight). Notes: Coefficients from linear regression are reported, the bar shows 95% CI. All models adjust for the confounders and district fixed effects reported in Table 2. BW is reported in grams. SCST are scheduled caste and scheduled tribes which are socially and economically disadvantaged communities.

We further examined how the effects of the pandemic on birth outcomes varied by maternal education, household caste and religion, and household wealth (Figs. 1 and 2). Figure 1 shows the results for birthweight, while Fig. 2 shows the results for LBW. The detailed regression results are reported in Supplementary Tables S2–S4. Overall, none of the coefficients in Fig. 1 are statistically significant except for the non-Hindu sample, indicating a heterogeneous association between the pandemic and birth weight according to the religion of the household. According to the models stratified by maternal education (Supplementary Table S2), the estimates of *β* in columns 1 and 2 for the pandemic cohort are not statistically significant, indicating that the association between BW and the number of COVID-19 pandemic years does not vary by maternal education. In the non-Hindu sample, infants born during the pandemic had 35 g lower BW than those born before the pandemic (col 4, Supplementary Table S4).

Figure 2 shows the associations between LBW and pandemic exposure for the subgroups mentioned above. The results for LBW were different from those for BW. We found significant heterogeneity in the associations between LBW and household and maternal characteristics. Of the nine subsamples, the association is statistically significant in eight samples except for the middle wealth groups. Compared with the subsample with secondary maternal education (schooling > 5 years), the pandemic cohorts born to mothers with primary education or less than primary education (schooling 5 years) had slightly greater odds of having LBW (Supplementary Table S2). Furthermore, in the less than primary schooling sample, the adjusted OR (aOR) for LBW was 1.09 (95% CI: 1.00–1.19), while the odds of LBW were 1.06 higher for pandemic cohorts born to mothers with secondary and higher education (95% CI: 1.01–1.12). There was no significant difference in the association between births during the pandemic and LBW according to household wealth group (Supplementary Table S3). The aORs for LBW were greater for infants born to SCST households (1.09 vs. 1.07) and non-Hindu households (1.16 vs. 1.07) than for infants born to non-SCST and Hindu households, respectively (Supplementary Table S4).

### Robustness checks

Table 3 examines the robustness of our main results to alternative definitions of pre-pandemic cohorts. One might worry that COVID-19 lockdowns in India were imposed on 24th March 2020, so births in Jan–Feb 2020 should be included in non-pandemic cohorts. Given the low infection rate and slow spread of COVID-19 in the first two months of 2020, it is unlikely that households may have changed their behavior in response to COVID-19 infections. Therefore, birth outcomes in the months of January and February 2020 are unlikely to be affected by COVID-19 and thus should be part of pre-pandemic cohorts. To formally investigate this, we sequentially included births in January and March 2020 in the pre-pandemic group. The results in Table 3 show that our results do not significantly change when we include children born in January and February 2020 in the pre-pandemic cohorts (columns 1–2). In columns 3–4, we include children born in Jan–March 2020 in the pre-pandemic cohorts. All the results in Table 3 are qualitatively similar to our main findings. Finally, we adjusted for pregnancy duration, and our results were robust to the inclusion of this variable (results available on request).

**Table 3:** Robustness checks and sensitivity analyses (fixed effects models)

	BW	LBW	BW	LBW
	(β)	AOR (95% CI)	(β)	AOR (95% CI)
	(1)	(2)	(3)	(4)
Pandemic cohort	−10.93**	1.08	−11.69	1.08
	(5.50)	(1.03–1.15)	(5.49)	(1.02–1.14)
Controls	Yes	Yes	Yes	Yes
District fixed effects	Yes	Yes	Yes	Yes
*N*	207,893	207,893	208,950	208,950

Notes: CI confidence intervals, AOR adjusted odds ratios, BW birthweight, LBW low birth weight; LBW (birthweight <2500 g). Robust standard errors are reported in parentheses in columns, while 95% CIs are reported in parentheses in column 2. Controls: gender, birth order, caste. Religion, mother’s age, rural, mother’s education, and wealth index. The non-pandemic cohorts include children born from July 2014 to Feb 2020 in columns 1–2 and from July 2014 to March 2020 in columns 3–4. The pandemic group includes children born from April 2020 to April 2021. ** denotes statistical significance at the 5% level of significance.

## Discussion

Using nationally representative data, we investigate the prevalence of LBW during the COVID-19 pandemic in India. The nationwide lockdown on 24th March 2020 provided a unique opportunity to compare the prevalence of BW and LBW among infants born during the pandemic and pre-pandemic periods. To the best of our knowledge, this study is one of the largest and most comprehensive studies to examine the impacts of the pandemic on BW and LBW incidence in a setting with poor health infrastructure and a high incidence of LBW. The principal finding of our study is that BW decreased by 11 g and that the odds of LBW increased during the COVID-19 pandemic.

Previous studies have shown conflicting effects of the pandemic on BW and LBW. Some studies reported an increase in BW, while other studies reported either no evidence of changes in BW or a decrease in BW. For example, prior studies revealed a significant increase in birthweight among term infants during the pandemic in South Korea^15^, Austria^18^, and China^19^. In contrast, a study conducted in the USA revealed that the pandemic (March 1 to December 3, 2020) was associated with a greater risk of poor fetal growth but was not associated with stillbirths^20^. A meta-analysis revealed a statistically nonsignificant difference in the risk of LBW between births in the pandemic and pre-pandemic periods^21–23^. Notably, a comprehensive study of births in developing countries, including Kenya, Zambia, the Democratic Republic of the Congo, Pakistan, India, and Guatemala, did not provide evidence of adverse impacts on LBW during the COVID-19 period compared with previous years^24^.

Several mechanisms may be responsible for our findings. One is the direct impact of the SARS-CoV-2 virus on the health of pregnant mothers. Direct infection of pregnant mothers may have contributed to their infants being born with lower BW. Other mechanisms are indirect. For example, the stress of the pandemic and its disruptions to the economy may have adversely impacted the health of expecting mothers. Nonpharmaceutical interventions such as formal or informal lockdowns and social distancing may also have created stresses for households either due to increased isolation or through their impact on labor markets. Other indirect channels involve the disruption of maternal and neonatal services. Due to the lockdown, prenatal services were likely affected, and women were reluctant to use health facilities for maternal and neonatal care in Nepal^25^. Several prior studies have shown that the use of prenatal care has positive impacts on BW in Mexico^26^ and India^27^.

It is important to recognize that the types of mothers who gave birth during the pandemic may have had characteristics that are also associated with lower BW. Although we cannot completely rule out selection bias of this nature, we do point out that although Table 1 shows some significant differences between the pandemic and pre-pandemic cohorts, they are not systematically different; for example, we show that the poorest and the wealthiest mothers were both more likely to give birth during the pandemic. Moreover, we adjusted for observable confounding variables in some of our analyses, and we still demonstrated that the pandemic cohort included infants with lower BW and a greater likelihood of LBW.

Furthermore, isolating mechanisms are critical not only for our understanding of the current COVID-19 response but also for understanding how we should approach future pandemics. If the direct effects of SARS-CoV-2 are responsible for the lower BW for pandemic-born infants, then careful control of the virus would improve infant health. If the mechanism is indirect, then careful attention will need to be given to ways of mitigating any adverse impacts of the pandemic response while still controlling viral spread. Given our data, we cannot isolate which of these mechanisms is most responsible for our findings.

Despite this, some insights can be gleaned from the experiences of China and South Korea, which experienced minimal circulation of SARS-CoV-2 during this time. A study from China showed that infants born during the pandemic in Nanjing Province, despite its zero COVID-19 policy, had lower BW and a greater prevalence of LBW than infants born before the pandemic^14^. The authors calculated an adjusted OR for LBW of 1.13, which is *greater* than our adjusted OR for LBW of 1.08. However, a similar study estimated a marginal decrease in BW of 1.27 g in a fully adjusted model, and they also reported that the odds of preterm birth and LBW declined during the pandemic^15^. Although there are no stark takeaways from these two case studies, they do illustrate that BW declined during the pandemic in two scenarios with limited viral circulation, suggesting that the indirect effects of the pandemic may be important factors.

Although this study is the first to analyze the effects of the SARS-CoV-2 pandemic on infant outcomes in India using a nationally representative sample, it has a few limitations. First, this was an observational study, and therefore, we were not able to have an adequate control group due to the sweeping nature of global pandemics. This limits our ability to make any causal interpretations of our estimated results. However, the high-quality data we used, the large sample size, and our ability to adjust for several important confounding factors lend credence to our finding that the COVID-19 pandemic is associated with a higher incidence of LBW in India. Second, the NFHS survey did not collect data on maternal infection with COVID-19. None of the sampled women in the NFHS were tested for COVID-19, so the prevalence of COVID-19 among the study population is unknown. COVID-19 infection during pregnancy could have affected BW through biological channels, while our study indirectly revealed the importance of behavioral channels, possibly through reduced access to and use of maternal and prenatal services during the pandemic^28^. Reduced prenatal care has been associated with a greater prevalence of LBW among socially and economically vulnerable populations^25,27^. Third, although our study revealed an increase in LBW during the pandemic, the mechanisms through which BW and LBW are affected are not fully understood and need to be further investigated. Fourth, we did not assess the impacts of COVID-19 exposure by trimester. Exposure to stressful events in the first trimester has been shown to have greater effects on birth weight than maternal stress in the second and third trimesters^29^. The NFHS survey has information on pregnancy duration in months ranging from 4–10 months, and future research should examine the effects by trimester. As a robustness check, we adjusted for pregnancy duration, and the results were qualitatively similar.

Fifth, the birth weight data were collected either from the medical records (birth cards) or the mothers’ recalls. Mothers’ recall could be a source of recall bias or measurement error. Although prevailing evidence suggests that recall bias in BW is not very high in rich countries, there is evidence that these biases may be more important in middle-income countries^16^. Additionally, it is possible that pandemic and pre-pandemic women may have answered birth weight questions differently, which may bias our results. There is no evidence to suggest that this is the case, as we could not locate any studies that compared recall biases during and before the pandemic. Therefore, our study is unable to eliminate the possibility of recall bias. Furthermore, our results may be driven by seasonal patterns if there is evidence of seasonal fluctuations in birth weight. A study in India explored the seasonal trends in birthweight in the eastern part of Maharashtra, and the authors did not find any evidence of seasonal variability in the birthweight of children^30^. Our analytical data do not show any seasonal variation in birth weight. Finally, the other limitation of our study is that we are unable to make any statements about the longer-term effects of the pandemic on child health at this point. As more waves of data become available, researchers should investigate how the pandemic cohort fared in early childhood and adolescence.

Despite these limitations, our study contributes to understanding the short-term impacts of health pandemics on birth outcomes and empirically illustrates the adverse impacts of the COVID-19 pandemic on birthweight in India.

### Supplementary information

Supplementary Information

Reporting Summary

## Data Availability

The NFHS-5 data is available for legitimate academic research through the DHS Program at https://dhsprogram.com/data/at. Registration is required for access to data.

## Code availability

Codes used to generate the tables and results are available from^31^. https://zenodo.org/records/11193263.
