# Supplementary material for: Effects of COVID-19 pandemic on low birth weight in a nationwide study in India
Source: Commun Med (Lond). 2024 Jun 14;4:118. doi: 10.1038/s43856-024-00545-4 (PMC11178855; doi:10.1038/s43856-024-00545-4)
Supplement: Supplementary file 1 — Supplementary Information [file 43856_2024_545_MOESM1_ESM.pdf]

Supplementary Information

Kumar et al. 2024. Effects of COVID-19 pandemic on low birth weight in a nationwide study in India

Table of Contents

Supplementary Table S1. Association between COVID-19 period and birthweight, n=208,950 (Random effects model)…………………………………………………..…2

Supplementary Table S2. Heterogeneous results by mother's education (District fixed effects)……………………………………………………………………………………3

Supplementary Table S3. Heterogeneous results by wealth index (District fixed effects) ………………………………………………………………………………….....…4

Supplementary Table S4. Heterogeneous results by caste and religion (District fixed effects) ……………………………………………………………………………………5

Table S1: Association between COVID-19 period and birthweight (Random effects model)

	BW<br>(1)	LBW<br>(2)
	(β)	AOR (95% CI)
Pandemic cohort	-12.21**	1.09
	(4.55)	(1.04-1.14)
Gender (female=0)	-62.70 ***	1.17
	(2.47)	(1.14-1.20)
Birth order	9.49 ***	0.97
	(1.70)	(0.95-0.98)
Scheduled caste/tribe	-1.29	0.98
	(3.54)	(0.95-1.01)
Hindu religion	-50.46 ***	1.14
	(4.81)	(1.09-1.19)
Rural	11.85***	0.94
	(4.20)	(0.90-0.97)
Mother's age	3.21***	0.98
	(0.38)	(0.98-0.99)
Mother's education		
No education	*Ref.*	*Ref.*
Primary	2.31	1.01
	(5.07)	(0.96-1.05)
Secondary	36.94***	0.89
	(5.07)	(0.86-0.93)
Higher	96.33***	0.73
	(5.87)	(0.69-0.77)
Wealth index		
Poorest	*Ref.*	*Ref.*
Poor	33.96***	0.89
	(4.02)	(0.86-0.92)
Middle	67.68***	0.78
	(4.74)	(0.74-0.81)
Rich	88.61	0.74
	(5.18)	(0.71-0.78)
Richest	112.90***	0.68
	(6.50)	(0.64-0.72)
Observations	208,950	208,950

Notes: CI: Confidence intervals; AOR: Adjusted Odds Ratios; BW: Birthweight; LBW: Low birth weight; LBW (birthweight < 2,500 grams). Pandemic cohorts are children born between April 2020 and April 2021. A few observations were omitted in column 2 because of all positive or negative outcomes in a district. ***, ** denotes significance at 1% and 5% level of significance, respectively.

Table S2: Heterogeneous results by mother's education (District fixed effects)

	BW	BW	LBW	LBW
	β	β	AOR (95% CI)	AOR (95% CI)
	(1) Mother's schooling ≤5 years	(2) Mother's schooling > 5 years	(3) Mother's schooling ≤ 5 years	(4) Mother's schooling > 5 years
Pandemic cohort	-14.38	-5.794	1.09	1.07
	(10.39)	(6.56)	(0.99-1.21)	(1.01-1.14)
Gender (female=0)	-55.48***	-65.98***	1.16	1.18
	(4.33)	(2.88)	(1.11-1.20]	(1.15-1.22)
Birth order	7.13***	9.19***	0.97	0.97
	(1.91)	(1.84)	(0.96-0.99)	(0.95-0.99)
Scheduled caste/tribe	-3.43	-12.43***	0.98	1.02
	(5.27)	(3.53)	(0.93-1.03)	(0.98-1.06)
Hindu religion	-55.68***	-20.91***	1.13	1.03
	(6.83)	(4.53)	(1.05-1.21)	(0.98-1.09)
Rural	10.16	11.21***	0.92	0.96
	(8.19)	(4.09)	(0.85-0.99)	(0.92-1.01)
Mother's age	2.08***	4.29***	0.99	0.98
	(0.537)	(0.39)	(0.98-0.99)	(0.98-0.99)
Wealth index				
Poorest	*Ref.*	*Ref.*	*Ref.*	*Ref.*
Poor	31.15***	36.77***	0.91	0.87
	(5.66)	(5.10)	(0.86-0.96)	(0.82-0.91)
Middle	61.33***	78.85***	0.77	0.75
	(7.45)	(5.35)	(0.72-0.83)	(0.71-0.80)
Rich	74.80***	110.23***	0.75	0.69
	(9.97)	(5.71)	(0.68-0.83)	(0.65-0.73)
Richest	92.45***	153.92***	0.67	0.59
	(15.15)	(6.59)	(0.58-0.77)	(0.55-0.64)
Observations	65,410	139,205	65,248	139,205

Notes: CI: Confidence intervals; AOR: Adjusted Odds Ratios; BW: Birthweight; LBW: Low birth weight; LBW (birthweight < 2,500 grams). Robust standard errors are reported in parentheses in columns 1-2, while 95% CI are reported in parentheses in columns 3-4. Pandemic cohorts are children born between April 2020-April 2021. Few observations were omitted because of all positive or all negative outcomes in column 3. ***, ** denotes statistical significance at 1% and 5% level of significance, respectively.

Table S3: Heterogeneous results by wealth index (District fixed effects)

	BW	BW	BW	LBW	LBW	LBW
	β	β	β	AOR (95% CI)	AOR (95% CI)	AOR (95% CI)
	(1) Poorest and poor	(2) Middle	(3) Rich and richest	(4) Poorest and poor	(5) Middle	(6) Rich and richest
Pandemic cohort	-8.05<br>(7.94)	-19.36<br>(12.91)	-11.98<br>(9.66)	1.08<br>(1.01- 1.17)	1.11<br>(0.97-1.24)	1.09<br>(0.98-1.21)
Gender (female=0)	-67.25***<br>(3.47)	-53.29***<br>(5.35)	-61.89***<br>(4.25)	1.19<br>(1.15- 1.23)	1.13<br>(1.08-1.20)	1.17<br>(1.12-1.22)
Birth order	7.38***<br>(1.71)	12.10***<br>(3.11)	10.68***<br>(2.91)	0.97<br>(0.95-0.99)	0.98<br>(0.95-1.01)	0.97<br>(0.94-1.00)
Scheduled caste/tribe	-11.04**<br>(4.13)	-10.63<br>(6.44)	-9.16<br>(5.54)	1.01<br>(0.97-1.05)	1.00<br>(0.94-1.07)	1.00<br>(0.95-1.06)
Hindu religion	-40.17***<br>(5.95)	-34.04***<br>(8.42)	-28.01***<br>(6.11)	1.11<br>(1.04- 1.18)	1.11<br>(1.02-1.22)	1.03<br>(0.96-1.10)
Rural	1.06<br>(8.22)	2.46<br>(7.70)	9.10*<br>(4.87)	1.01<br>(0.93-1.10)	0.96<br>(0.89-1.04)	0.95<br>(0.90-1.00)
Mother's age	3.80***<br>(0.46)	3.01***<br>(0.71)	2.08***<br>(0.57)	0.98<br>(0.98-0.99)	0.98<br>(0.98-0.99)	0.99<br>(0.99-1.00)
Mother's Education						
None	*Ref.*	*Ref.*	*Ref.*	*Ref.*	*Ref.*	*Ref.*
Primary	11.23**<br>(5.47)	-5.12<br>(11.46)	-33.74**<br>(13.61)	0.98<br>(0.93-1.04)	0.98<br>(0.88-1.09)	1.14<br>(1.01-1.28)

Secondary	41.93***	25.86**	26.58**	0.87	0.91	0.94
	(4.61)	(9.36)	(10.53)	(0.84-0.91)	(0.83-1.00)	(0.84-1.04)
Higher	90.66***	87.76***	91.75***	0.78	0.77	0.74
	(10.51)	(12.05)	(11.06)	(0.71-0.86)	(0.67-0.87)	(0.66-0.83)
Observations	97,655	41,250	65,710	97,558	40,980	65,187

Notes: CI: Confidence intervals; AOR: Adjusted Odds Ratios; BW: Birthweight; LBW: Low birth weight; LBW (birthweight < 2,500 grams). Robust standard errors are reported in parentheses in columns 1-3, while 95% CI are reported in parentheses in columns 4-6. Pandemic cohorts are children born between April 2020-April 2021. A few observations were omitted in columns 4-6 because of all positive or negative outcomes in a district. ***, **, * denotes statistical significance at 1%, 5%, and 10% level of significance.

Table S4: Heterogeneous results by caste and religion (District fixed effects)

	BW	BW	BW	BW	LBW	LBW	LBW	LBW
	β	β	β	β	AOR (95% CI)	AOR (95% CI)	AOR (95% CI)	AOR (95% CI)
	(1) SC/ST	(2) Other Caste	(3) Hindu	(4) Non-Hindu	(5) SC/ST	(6) Other Caste	(7) Hindu	(8) Non-Hindu
Pandemic cohort	-12.79	-10.14	-6.56	-34.58**	1.09	1.08	1.07	1.19
	(8.32)	(7.40)	(6.06)	(13.50)	(0.99-1.18)	(1.02-1.15)	(1.01-1.13)	(1.03-1.37)
Gender (female=0)	-60.33***	-64.19***	-64.61***	-56.72***	1.17	1.17	1.18	1.13
	(3.75)	(3.12)	(2.79)	(4.73)	(1.13-1.22)	(1.14-1.20)	(1.15-1.21)	(1.08-1.19)
Birth order	11.66***	8.24***	8.84***	10.51***	0.95	0.97	0.97	0.95
	(1.89)	(1.84)	(1.62)	(2.28)	(0.93-0.97)	(0.95-0.99)	(0.95-0.98)	(0.93-0.98)
Scheduled caste/tribe	--	--	-8.68***	-9.99	--	--	1.01	0.97
			(3.17)	(8.71)			(0.97-1.04)	(0.89-1.07)
Hindu religion	-27.83***	-35.79***	--	--	0.10	1.07	--	--
	(8.05)	(4.46)			(1.01-1.20)	(1.02-1.13)		
Rural	13.14**	10.19**	12.88***	2.97	0.96	0.93	0.93	1.00
	(6.45)	(4.48)	(4.38)	(7.04)	(0.90-1.03)	(0.89-0.98)	(0.89-0.98)	(0.91-1.09)
Mother's age	3.27***	2.65***	3.22***	2.26***	0.99	0.99	0.98	1.00
	(0.48)	(0.42)	(0.38)	(0.57)	(0.95-0.99)	(0.98-0.99)	(0.98-0.99)	(0.99-1.00)
Mother's Education								
None	*Ref.*	*Ref.*	*Ref.*	*Ref.*	*Ref.*	*Ref.*	*Ref.*	*Ref.*
Primary	7.29	-6.97	-4.13	10.34	1.01	1.01	1.03	0.96

	(6.57)	(6.47)	(5.41)	(8.86)	(0.95-1.09)	(0.96-1.08)	(0.98-1.08)	(0.88-1.05)
Secondary	32.57***	30.20***	35.03***	23.59***	0.92	0.91	0.90	0.93
	(5.58)	(5.25)	(4.44)	(7.59)	(0.87-0.98)	(0.86-0.95)	(0.86-0.94)	(0.86-1.01)
Higher	73.30***	90.81***	87.72***	85.59***	0.83	0.73	0.76	0.74
	(8.70)	(6.69)	(5.96)	(10.88)	(0.76-0.91)	(0.68-0.78)	(0.71-81)	(0.66-0.84)
Wealth index								
Poorest	*Ref.*	*Ref.*	*Ref.*	*Ref.*	*Ref.*	*Ref.*	*Ref.*	*Ref.*
Poor	31.32***	34.65***	34.66***	28.98***	0.91	0.87	0.89	0.86
	(5.29)	(5.38)	(4.34)	(7.45)	(0.86-0.95)	(0.83-0.91)	(0.86-0.93)	(0.79-0.93)
Middle	65.69***	71.52***	73.41***	52.33***	0.78	0.76	0.76	0.79
	(6.39)	(5.79)	(4.90)	(8.54)	(0.73-0.83)	(0.71-0.80)	(0.72-0.80)	(0.71-0.86)
Rich	98.82***	90.79***	96.73***	79.41 ***	0.70	0.73	0.71	0.74
	(7.84)	(6.27)	(5.52)	(9.71)	(0.64-0.76)	(0.68-0.77)	(0.68-0.75)	(0.67-0.83)
Richest	132.26***	119.98***	131.16***	103.01***	0.65	0.65	0.63	0.66
	(10.67)	(7.36)	(6.81)	(11.67)	(0.58-0.72)	(0.60-0.70)	(0.59-0.68)	(0.58-0.76)
Observations	80,968	123,647	152,740	51,875	80,939	123,524	152,655	51,571

Notes: CI: Confidence intervals; AOR: Adjusted Odds Ratios; BW: Birthweight; LBW: Low birth weight; LBW (birthweight < 2,500 grams). Robust standard errors are reported in parentheses in columns 1-4, while 95% CI are reported in parentheses in columns 5-8. Pandemic cohorts are children born between April 2020-April 2021. All columns include district fixed effects. SC/ST are scheduled caste and scheduled tribes which are socially and economically disadvantaged communities. ***, **, * denotes statistical significance at 1%, 5%, and 10% level of significance.
